# *EHP* Classic Paper of the Year, 2011

**DOI:** 10.1289/ehp.1103844

**Published:** 2011-06

**Authors:** Hugh A. Tilson

**Affiliations:** Editor-in-Chief, *EHP*, E-mail: tilsonha@niehs.nih.gov

*Environmental Health Perspectives* (*EHP*) established the Paper of the Year in 2008 (Tilson 2008) as a way of highlighting high-quality articles published in the journal. Until now, the Paper of the Year for any given year was selected on the basis of citations received over the preceding 60 months. Starting this year, this award will be known as the *EHP* Classic Paper of the Year. This award will be given to the most highly cited Research Article, Commentary, or Review Article over the preceding 60 months. A new award, the *EHP* Paper of the Year, will honor a Research Article published in the preceding year. Final selection of both awards will be subject to approval by the *EHP* Board of Associate Editors. The winner of the *EHP* Paper of the Year will be announced later in the calendar year.

We are proud to announce that the 2011 *EHP* Classic Paper of the Year is “Maternal Genistein Alters Coat Color and Protects *A^vy^* Mouse Offspring from Obesity by Modifying the Fetal Epigenome” by Dana C. Dolinoy, Jennifer R. Weidman, Robert A. Waterland, and Randy L. Jirtle. This article was published in the April 2006 issue of *EHP* (Dolinoy et al. 2006) and has been cited more than 35 times per year since it was published.

At the time the paper was written, it was increasingly recognized that exposure to nutritional, chemical, and behavioral factors could alter gene expression and affect health and disease not only by mutating promoter and coding regions of genes but also by modifying the epigenome. The epigenome comprises the heritable changes in gene expression that occur in the absence of changes to the DNA sequence itself, including DNA methylation and chromatin packaging. If the genome is compared to the hardware in a computer, the epigenome is the software that directs the computer’s operation. Dolinoy et al. (2006) argued that identifying epigenetic targets and defining how they are influenced by nutrition and the environment might lead to the development of innovative diagnostic, treatment, and prevention strategies that target the “epigenomic software” rather than the “genomic hardware.”

Dolinoy et al. (2006) used the viable yellow agouti (*A^vy^*) mouse as an epigenetic biosensor to demonstrate that genistein, the major phytoestrogen in soy, increases DNA methylation of the *Agouti* gene, resulting in population-level decreased incidence of adult-onset obesity, diabetes, and cancer. At the inception of this study, epidemiological data suggested that increased dietary genistein plays a role in decreased incidence of cancer in Asians compared with Westerners. Thus, they hypothesized that early dietary exposures, including genistein, might be linked to adult health status via epigenetic mechanisms. These authors demonstrated that maternal dietary genistein supplementation of mice during gestation at levels comparable to humans consuming high soy diets shifted the coat color of heterozygous viable yellow agouti (*A^vy^/a*) offspring toward pseudoagouti. This phenotypic change was significantly associated with increased methylation of six cytosine–guanine sites in a retrotransposon upstream of the transcription start site of the *Agouti* gene. A significant finding was that genistein-induced hypermethylation persisted into adulthood, decreasing ectopic *Agouti* expression and protecting offspring from adult-onset obesity.

Dolinoy et al. (2006) provided the first evidence that *in utero* dietary genistein affects gene expression and alters susceptibility to obesity in adulthood by permanently altering the epigenome. They also established the framework for future studies by showing that both genistein and methyl donors, such as folic acid, betaine, and choline, counteract DNA hypomethylation caused by bisphenol A, an endocrine-active agent used to make polycarbonate plastic. Their results showed that simple dietary changes can protect against the deleterious effects of environmental toxicants on the fetal epigenome (Dolinoy et al. 2007).

*EHP* congratulates Dolinoy and colleagues for their contribution to the environmental health science literature. In addition to demonstrating that single-nucleotide polymorphisms can affect environmentally responsive genes, they demonstrated that early nutritionally and environmentally induced epigenetic modifications may be an alternative mechanism underlying individual susceptibilities to environmental agents.

## Figures and Tables

**Figure:**
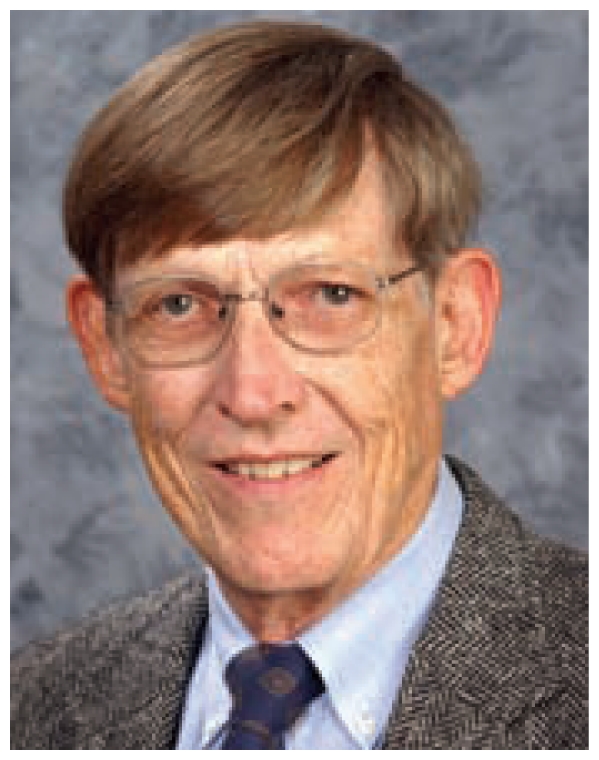
Hugh A. Tilson
